# Merits photocatalytic activity of rGO/zinc copper ferrite magnetic nanocatalyst for photodegradation of methylene blue (MB) dye

**DOI:** 10.1186/s11671-024-04162-x

**Published:** 2025-01-04

**Authors:** Osama H. Abuzeyad, Ahmed M. El-Khawaga, Hesham Tantawy, Mohamed Gobara, Mohamed A. Elsayed

**Affiliations:** 1 Department of Chemical Engineering, Military Technical College (MTC), Cairo, Egypt; 2 Department of Basic Medical Sciences, Faculty of Medicine, Galala University, Galala City, Suez 43511 Egypt

**Keywords:** Nanocomposites, Wastewater treatment, Methylene blue (MB), Photocatalysis

## Abstract

The world is now facing a water scarcity crisis due to waste, pollution, and uneven distribution of freshwater resources, which are limited. Thus, the creation of innovative, economical, and effective methods for purifying water is crucial. Here, the photo-assisted degradation of methylene blue (MB) dye under visible light and UV was achieved by using RGO photocatalyst loaded with Zn_0.5_Cu_0.5_Fe_2_O_4_ in three different loaded 10%, 20%, and 30% called MRGO 10, MRGO 20, and MRGO 30. Furthermore, all prepared samples was characterized by X-ray diffraction (XRD), fourier transformation infrared (FTIR), transmission electron microscope (TEM), vibrating sample magnetometer (VSM) and Raman analysis. After 40 min, the high photocatalytic efficacy effectively eliminated about 95.2% of the 10 ppm MB using 20 mg of MRGO 20 NPs at pH9 Visible light. From the results, the photocatalytic activity of MRGO 20 reduced to 54.6% after five cycles of methylene blue (MB) dye degradation. The produced samples' observed efficacy in both UV and visible light may encourage continued research into more effective photocatalysts for the filtration of water.

## Introduction

These days, one of the primary areas of focus for humans is the preservation and protection of natural resources. Regrettably, due to industrial and agricultural pollutants, water, the most vital resource for life, is the most contaminated natural resource [1]. High levels of pesticides, heavy metals, and dyes, among other contaminants, are present in contaminated water. Developing nations' economies benefit greatly from the textile and dyeing industries. According to research by the World Health Organization (WHO), the dyeing industries account for between 17 and 20 percent of all water pollution [2]. Many health problems have been related to these dye pollutions, such as irritation of the skin, problems with breathing, sweating excessively, cancer and nausea. Methylene Blue (MB) is an organic material that belongs to the cationic azo dye family, and it is inexpensive material. Methylene Blue (MB) is primarily utilized in ink industries and medication as well as in research facilities [3]. Traditional approaches to water treatment like coagulation, flocculation, sedimentation, and filtration, have been employed [4]. However, these methods often fall short in terms of efficiency and cost-effectiveness. Photocatalytic decomposition process is one of the most applicable processes that have been utilized for industrial dyes degradation [5, 6]. When exposed to sunrays or ultraviolet radiation, some of the dye's valence band electrons undergo photocatalytic degradation, moving into the conduction band. Consequently, the catalyst's surface developed holes and electrons. The hydroxide free radicals that the holes and electrons produced break down the dyes into gases that are not toxic like CO_2_ and H_2_O [7, 8]. Spinel magnetic ferrites have been the subject of several studies to date on the treatment of wastewater containing dyes [9, 10]. Its unique properties which include easy reparability from an external magnetic field, great abundance, highly reactive, cost-effective, non-toxic, and adequate photochemical stability, make it sensitive to visible light and a prime candidate for use as a catalyst in photodegradation processes to rid of organic compounds and toxic colours from water [11, 12]. Additionally, rGO was identified as a two-dimensional structured zero-bandgap semiconductor carbon network, a perfect surface for assembling nanoparticles onto its surface layers to create rGO-based nanocomposites [13, 14]. Because of the rGO material's high surface area (about 2630 m^2^ g^−1^), non-aggregation, increased light-absorption range, and significantly lower electron-hole pair recombination, the resulting rGO-based nanocomposites function as effective catalysts [15]. Various rGO-based hybrids containing metal and metal oxide nanoparticles are employed in photocatalysis applications these days [4, 16]. It has been created using a variety of techniques. When exposed to visible solar radiation, the material resulting from the nanocomposites has good properties for photocatalysis [17]. The rGO/ZCF nanocomposite was produced in this study using the co-precipitation technique and investigate its optical, structural, and photodegradation efficiency [18]. Additionally, to look at the suitability of removing Methylene Blue (MB) from aqueous systems. The combination of reduced graphene oxide (rGO) and zinc copper ferrite magnetic nanoparticles exhibits several advantages for the photocatalytic degradation of methylene blue (MB) dye.

## Materials and methods

### Chemicals

Fe_2_ (SO_4_)_3_·5H_2_O, Cu (SO_4_) ·6H_2_O, Zn (SO_4_) ·7H_2_O, NaOH, Graphite powder 99.5%, KMnO_4_ 98%, H_2_SO_4_ 98%, HCl 36%, H_2_O_2_ 35%, C₆H₈O₆ and MB were obtained from E-Merck Products. The remainder of the substances were not purified before use and had a purity comparable to analytical grade.

### Synthesis of rGO and rGO/ZCF nanocomposite

**Fig. 1:**
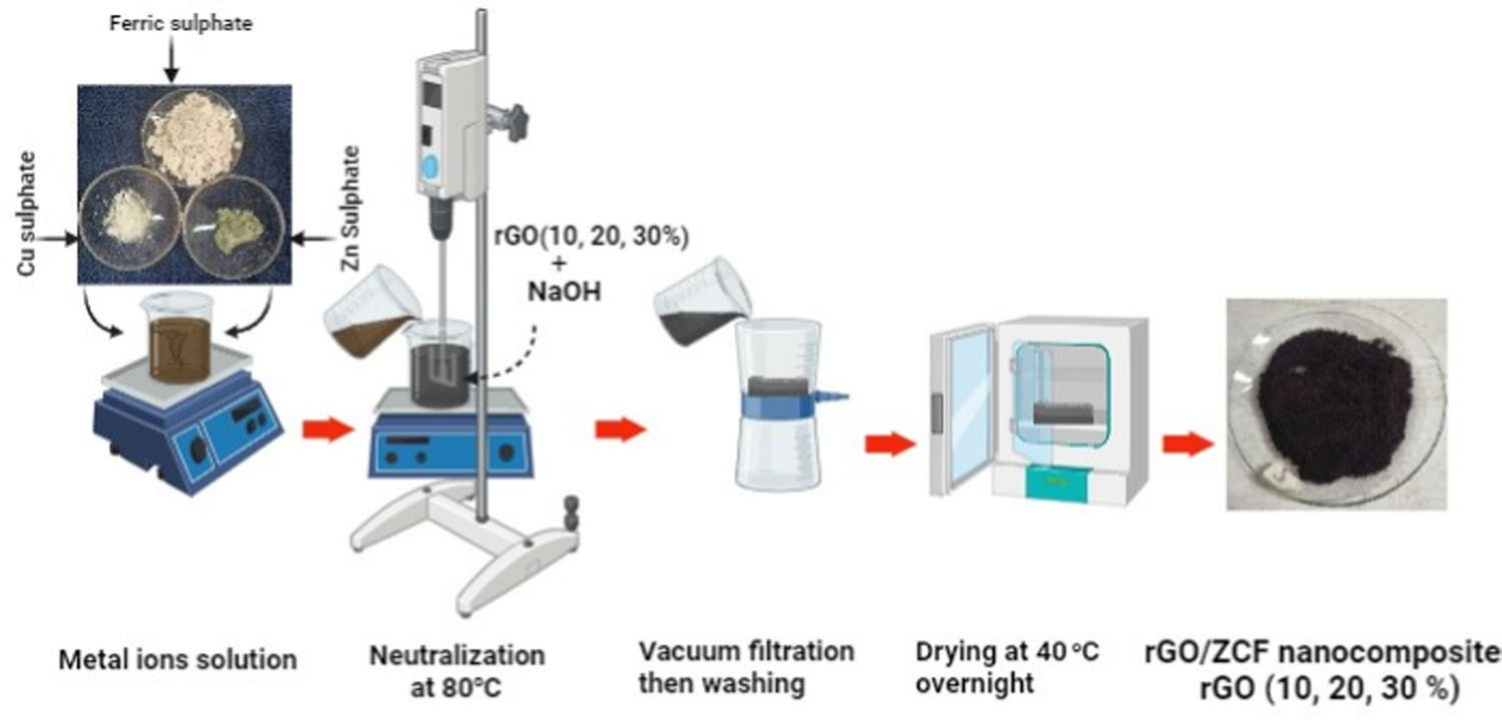
Schematic figure of rGO/ZCF Nanocomposite preparation

The modified Hummers' technique (R), addressed elsewhere, was used to synthesise RGO [19]. The rGO/ZCF Nanocomposite were produced via the co-precipitation technique. Fleetingly, the stoichiometric ratios of reagent grade Fe_2_(SO_4_)_3_·5H_2_O, Cu(SO_4_)·6H_2_O, and Zn(SO_4_)·7H_2_O were first thoroughly stirred for 30 min in 50 millilitres of DI water to achieve total solubility before it was poured into 1000 mL of 1.5 mol/L NaOH/rGO, containing varying rGO weight percentages 10%, 20%, and 30% solutions, and mechanically stirred at 100^◦^C for 1 h. The black precipitates were collected by magnetic separation and then washed thrice with ethanol and DI water. Afterwards, the synthesized nanocomposites were allowed to dry overnight at 40 °C in a vacuum oven [20]. The rGO obtained coated with Zn_0.5_Cu_0.5_Fe_2_O_4_ has been labelled as follows based on the weight percentage of rGO: Zn_0.5_Cu_0.5_Fe_2_O_4_, MRGO10, MRGO20, and MRGO30. As seen in Fig. 1, the samples were then sterilised with acetone and allowed to dry for 72 h at room temperature [21].

### Characterization of the rGO/ZCF nanocomposite

The rGO/ZCF nanocomposite that was produced was identified using several characterization techniques. For morphological characterisation [22], a High-Resolution Transmission Electron Microscope (HRTEM) images were obtained using a JEOL JEM1010 transmission electron microscope equipped with a CCD camera and an acceleration voltage of 100 kV [23]. X-ray diffraction (XRD) spectra were obtained using an X-ray diffraction (XRD) a Siemens D5000 powder X-ray diffractometer (Siemens, Houston, TX, USA) with Cu Ka radiation (λ = 1.5418A°) was used to identify and examine the phase of the Cu Zn ferrite nanopowders. Spectroscopic analysis was carried out on continuous collections of Raman spectra with a spectral resolution of 4 cm^−1^ [24]. The Raman excitation source was focussed using a Nikon 20 objective (10 mW, 532 nm neodymium-doped yttrium aluminium garnet (Nd:YAG) laser-Bruker, Germany) [25]. The data collection time for the 50 × 50 μm diaphragm illumination zone was 1000 ms (co addition 3). Fourier transform infrared analysis (FTIR) was utilized. The JASKO 4100 spectrometer (Japan) (Jasko, Tokyo, Japan) was used to carry out FTIR analysis with a resolution of 4 cm^−1^ in the 500–4000 cm^−1^ range [26]. The magnetic characteristics (saturation magnetization Ms, magnetic remanence Mr, and coercivityHc) of magnetite nanoparticles MRGO 10, MRGO 20, and MRGO 30 were measured at room temperature using the Hielscher UP200S vibrating sample magnetometer (VSM). The UV–Vis spectrum was analyzed with an Agilent Cary 60 UV–Vis spectrophotometer [27].

### Photocatalytic reactor

**Fig. 2:**
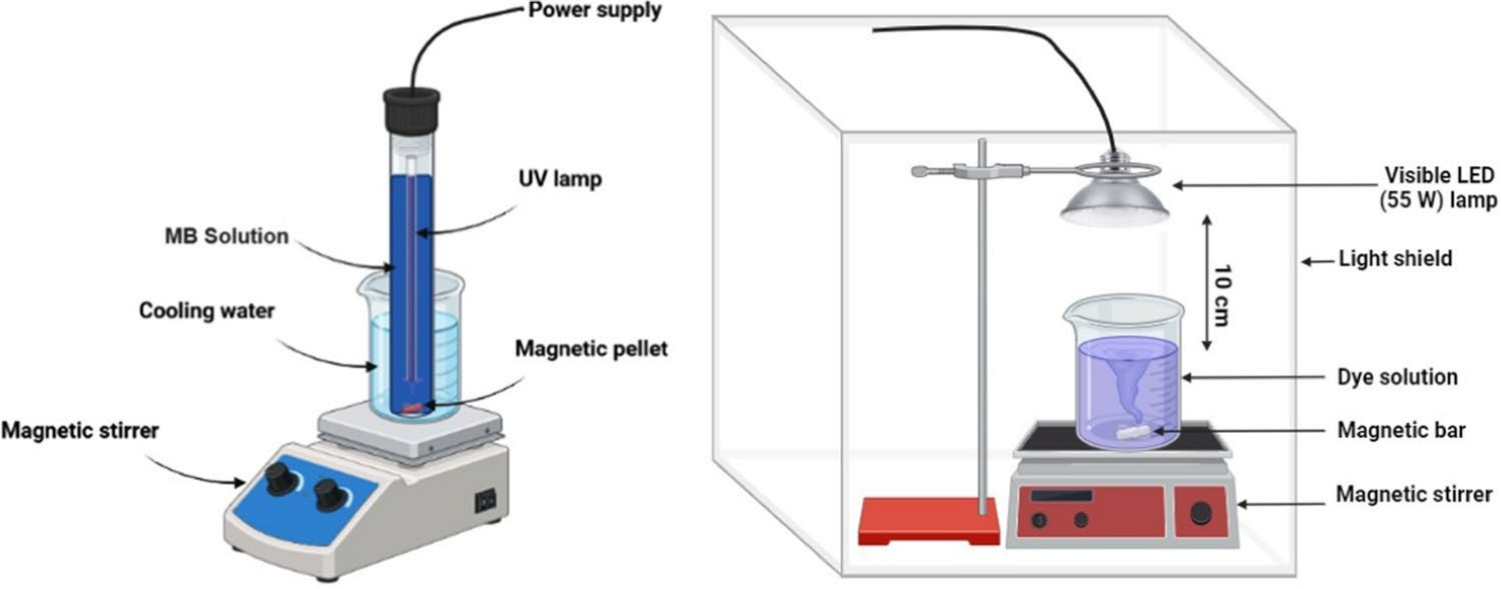
The photocatalytic setups for UV and Visible light irradiation

The (MB) was photocatalytically degraded using a UV light and a catalyst known as a rGO/ZCF nanocomposite [21]. As seen in Fig. 2, the UV reactor in use was a glass cylinder form (100 ml) with dimensions of 27 cm in length by 3 cm in diameter. It was also coated in a thin coating of aluminium foil. The Photocatalytic Reactor was loaded with 50 mL of MB dye solutions.

The Philips TUV 11WG11 T5, a high-pressure mercury lamp with a mean wavelength of 254 nm and an operating power of 11 W [28], was the commercial UV-C lamp used to irradiate the area. The photoreactor is submerged in the tainted solutions and maintained at a temperature of around 25 °C via a cold water bath. In order to reduce irradiation loss, a lamb made up of 48 white LEDs with an emission range of 400–800 nm and a nominal power of 48 W was used to provide visible light. The LEDs were encircled by aluminium reflectors. The light and reactor are set at a distance of 10 cm, with the irradiation applied from above.

Prior to inserting the UV irradiation, the pollutant MB dye and catalyst rGO/ZCF nanocomposite were placed inside the glass cylindrical reactor. A syringe was used to remove a 2 ml of the MB dye at a designated time, centrifuged after 20 min, and the absorbance was measured using a spectrophotometer at a wavelength of 465 nm [29]. Using the Eq. (1) below, the photodecomposition efficiency (Removal %) was determined:

1 $${\text{Removal \%}}=1-\left(\frac{Ct}{C0}\right)*100$$

where, Co is the starting concentration of the MB (ppm) and Ct is the concentration at time (t) of MB (ppm). The photocatalytic degradation's operating factors, such as pH and starting pollutant concentrations, were investigated [30].

## Results and discussion

### Characterization of rGO/ZCF Nanocomposite

#### High-resolution transmission electron microscope (TEM)

**Fig. 3:**
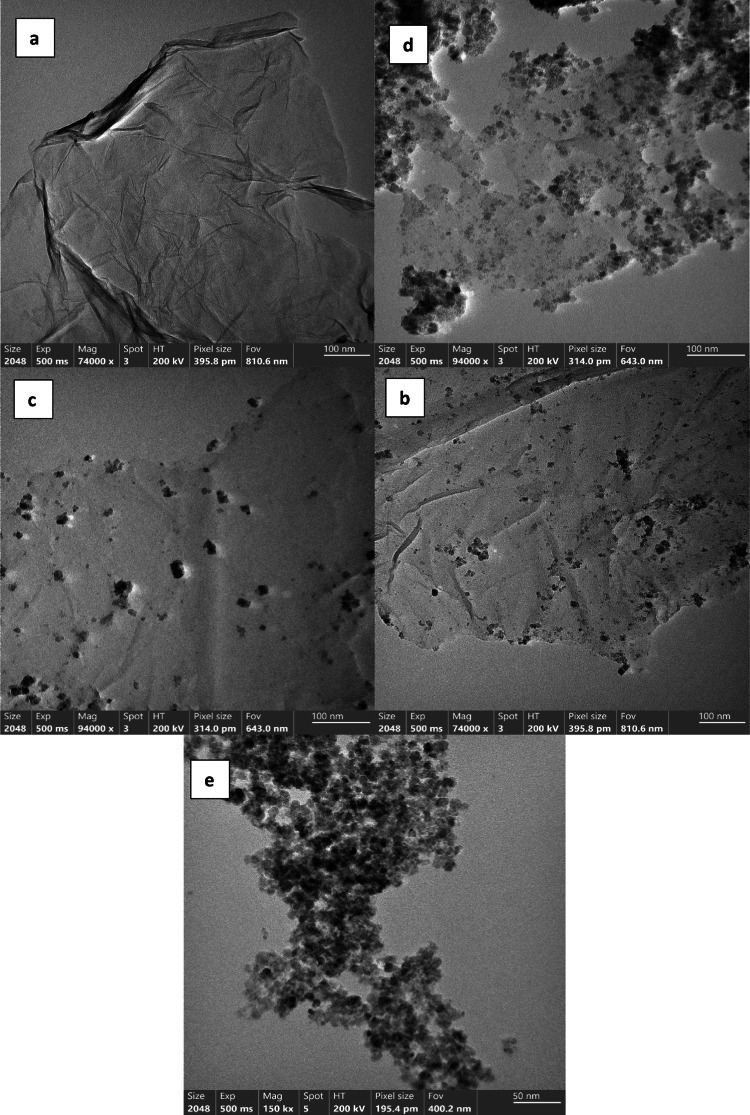
TEM images of the **a** rGO sheets, **b** MRGO10, **c** MRGO20, **d** MRGO30 and **e** Zn_0.5_Cu_0.5_Fe_2_O_4_

A transmission electron microscope (TEM) was utilized for further identification and confirmation of the particle morphology and size [31, 32]. The TEM pictures of the clusters of Zn_0.5_Cu_0.5_Fe_2_O_4_ nanoparticles, rGO, and Zn_0.5_Cu_0.5_Fe_2_O_4_/rGO mixed nanocomposites with varying rGO weight percentages, designated as MRGO10, MRGO20, and MRGO30, are shown in Fig. 3. In addition to displaying the decreased graphene oxide sheets' laminar characteristics (Fig. 3a). Furthermore, the transmission electron microscopy (TEM) pictures presented in Fig. 3b, c, and d highlight the effective creation of various hybrid nanocomposites with a consistent dispersion of Zn_0.5_Cu_0.5_Fe_2_O_4_ nanoparticles on rGO sheets, hence mitigating their unintended agglomerations [33]. The structural characterisation of the synthesised Zn_0.5_Cu_0.5_Fe_2_O_4_ is shown in Fig. 3e. Zn_0.5_Cu_0.5_Fe_2_O_4_ has an estimated dimension of 38 nm.

#### The X-ray diffraction

**Fig. 4:**
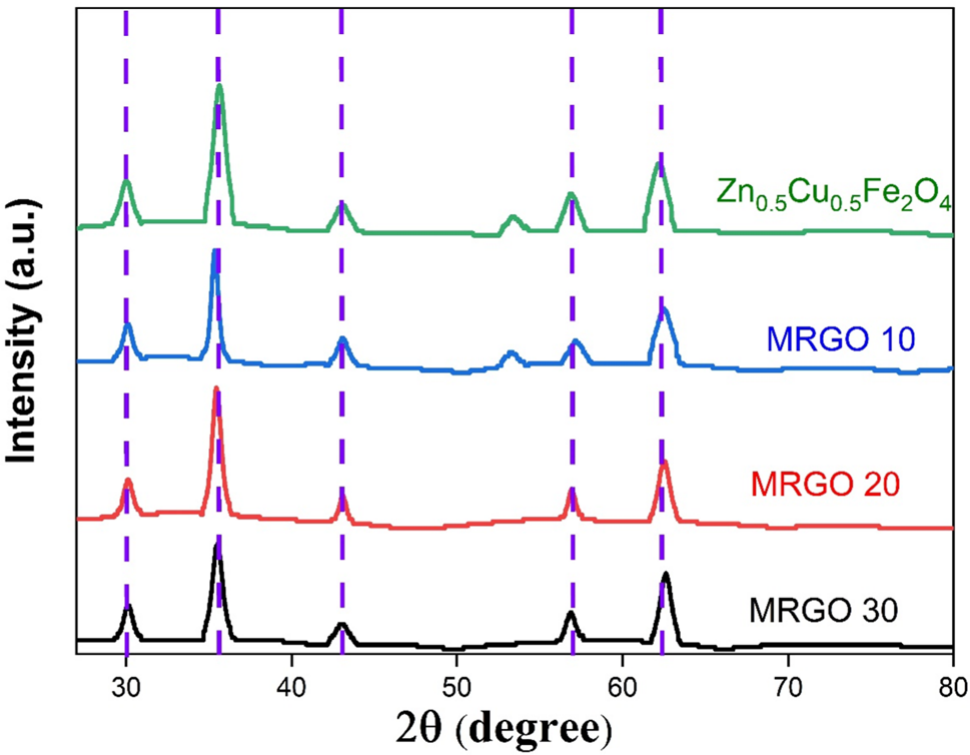
The XRD pattern spectra for the synthesized (a) Zn_0.5_Cu_0.5_Fe_2_O_4_ (green line), MRGO 10 (blue line), MRGO 20 (red line), and MRGO 30 (black line)

X-ray diffraction (XRD) was employed to examine crystalline structure, chemical structure of the prepared samples [34]. The XRD information attained for the rGO/ZCF nanocomposite with different rGO ratio (10, 20, 30%) coded MRGO10, MRGO 20 and MRGO 30, produced by co-precipitation exhibits varying crystallinity based on composition, as shown in Fig. 4. These samples' XRD examination revealed well defined diffracted peaks at 2θ = (31◦, 37◦, 43◦, 57◦, and 62◦), which correlate to (220, 311, 400, 511, and 440) [20, 29, 30]. These peaks indicated a spinel structure. For Zn_0.5_Cu_0.5_Fe_2_O_4_, the spinel structure peaks were sharper and more intense; however, when the rGO loading (wt%) increased, the sharpness and intensity dropped. This is explained by the fact that adding more rGO to the composite than Zn_0.5_Cu_0.5_Fe_2_O_4_ caused the crystallinity to drop. The strength of the fundamental cubic spinel ferrite's diffraction peak in the (311) plane, which is used to quantify the degree of crystallinity [35], corresponds to 2θ values ~ 35.2°, according to Scherrer's equation [36]. From the results, the highest crystalline domain size was observed for Zn_0.5_Cu_0.5_Fe_2_O_4_, MRGO10, MRGO 20 and MRGO 30 were 38.7, 29.3, 20.1, 10.8 nm, respectively. This attitude reveals the clear impact of an increase in the rGO loading content on lowering the crystallinity/order of the nanocomposites. On the other hand, the Crystallite size and crystallinity have been recognized as important parameters that influence the photocatalytic performance of the photocatalyst. It’s generally accepted that, with the decreases of diameter, the performances of adsorption and photocatalysis of the photocatalyst are improved [37]. Cheng et al. [32] found that the AgI/BiOI photocatalysts display size-dependent photocatalytic activity, which increases with the smaller size of the AgI NPs. This is believed to be related to the larger number of surface-active sites and faster spatial charge transfer.

#### FTIR analysis

**Fig. 5:**
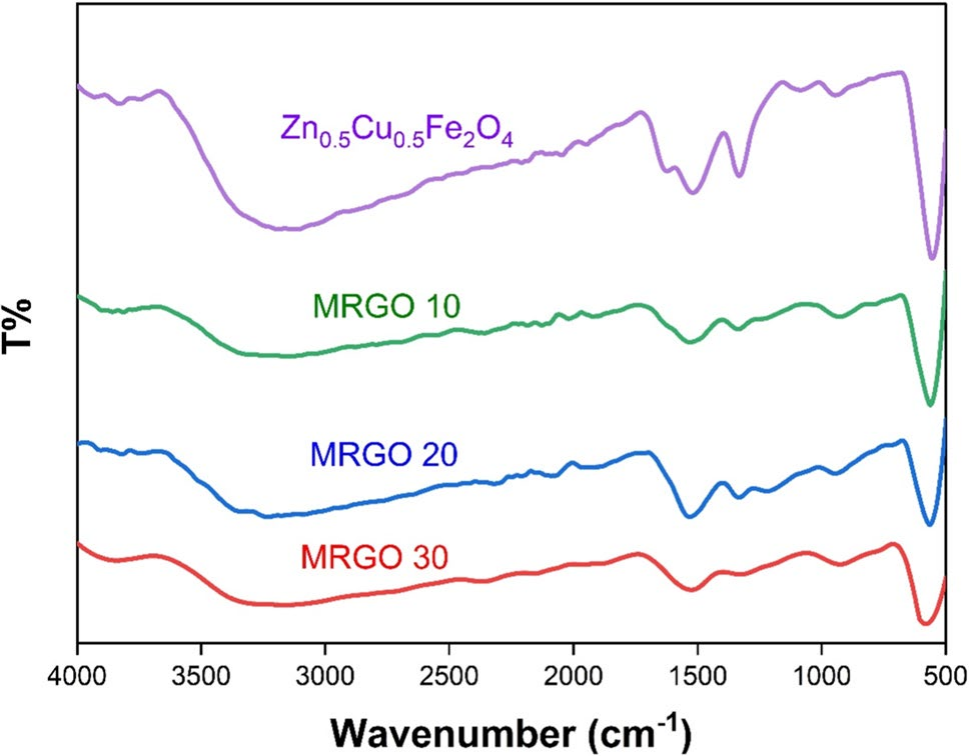
FTIR spectra of the synthesized rGO/ZCF Nanocomposite

Fourier-Transform Infrared (FTIR) provides information about molecular structure and chemical composition [38]. The FTIR spectra of the produced nanocomposites is displayed in Fig. 5. The resulting spectra may be broadly classified into two areas. The stretching band of lattice water H–O–H (3200–3600 cm^−1^) [39] and the fingerprint area (500–1800 cm^−1^) [40] are represented by the first and second, respectively. The detected peaks in the first area correspond to the characteristic features of a ferrospinel fingerprint [7]. The existence of rGO's aromatic rings was revealed by the presence of C = C stretching vibrations at 1588 cm^−1^ [41]. The C=C stretching vibrations play a crucial role in the photocatalytic process by enhancing light absorption, influencing charge dynamics, and increasing reactivity. Understanding these effects can aid in the design and optimization of photocatalytic materials for various applications, including environmental remediation and energy conversion [42, 43]. When the quantity of rGO increased, the distinctive peak of Zn_0.5_Cu_0.5_Fe_2_O_4_ dropped and occurred at 581 cm^−1^ for Fe single bond O single bond Fe vibration [44, 45].

#### Raman analysis

**Fig. 6:**
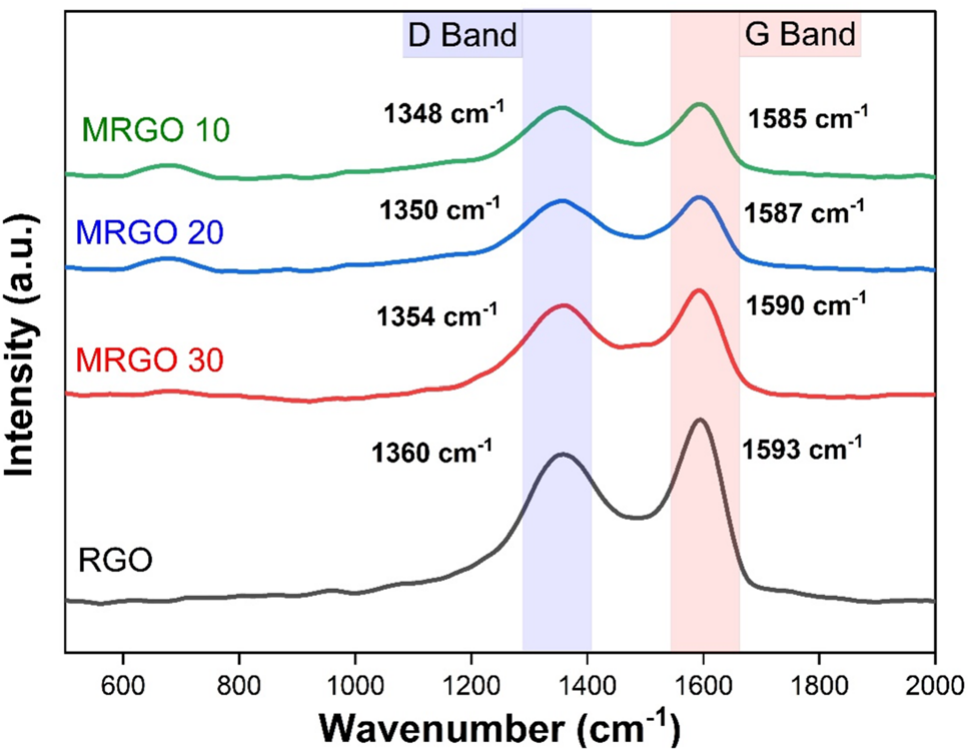
Raman spectra of the synthesized rGO/ZCF Nanocomposite

The Raman spectra of the Zn_0.5_Cu_0.5_Fe_2_O_4_@rGO and rGO composites (MRGO10, MRGO20, and MRGO30) are displayed in Fig. 6. The development of Zn_0.5_Cu_0.5_Fe_2_O_4_ in the obtained mixed nanocomposites was verified by the A1g mode presented at 668 cm^−1^ wave number for the Zn_0.5_Cu_0.5_Fe_2_O_4_@rGO composites [44]. In accordance with this, the rGO and Zn_0.5_Cu_0.5_Fe_2_O_4_@rGO composites can be clearly separated by their D and G bands. The G band identified at 1593 cm^−1^ in rGO causes in-plane breathing vibration of the sp^2^ carbon rings. The D band, observed at 1360 cm^−1^ for rGO is formed by the sp^2^ carbon lattice with sp^3^ deficiencies blowing out of the plane.

It was observed that the G and D band's intensity may be decreased by the Zn_0.5_Cu_0.5_Fe_2_O_4_ formations in the hybrid nanocomposites. Furthermore, a higher Zn_0.5_Cu_0.5_Fe_2_O_4_ concentration in the conducted nanocomposites may be linked to the observed rising shift for the mentioned G and D bands to the left (decreasing). This may be explained by the Zn_0.5_Cu_0.5_Fe_2_O_4_ nanoparticles arranging the carbon atoms in the rGO sheet and causing imperfections to appear in it. Furthermore, as seen in Fig. 6, The intensity ratios (IG/ID) of the G and D bands for rGO, MRGO 30, MRGO 20, and MRGO 10 were 1.05, 0.98, 0.97, and 0.96, respectively, as a result of this phenomena. Only the rGO (lower defects) composite had an IG/ID value more than (1); the other Zn_0.5_Cu_0.5_Fe_2_O_4_@rGO composites had values less than (1), indicating that defects in the composites grew as the amount of Zn_0.5_Cu_0.5_Fe_2_O_4_ combined with rGO sheets increased [46, 47].

#### VSM analysis

**Fig. 7:**
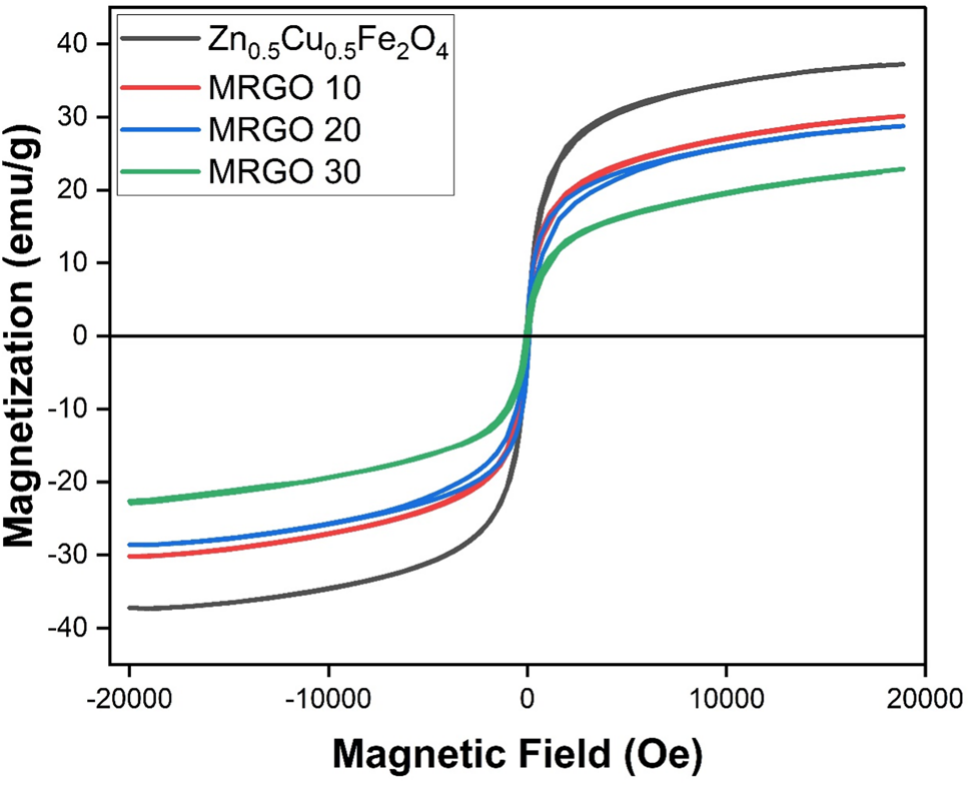
The hysteresis curves for Zn_0.5_Cu_0.5_Fe_2_O_4_, MRGO 10, MRGO 20, and MRGO 30

A vibrating sample magnetometer (VSM) measures the magnetic characteristics of Zn_0.5_Cu_0.5_Fe_2_O_4_@rGO composites (MRGO 10, MRGO 20, and MRGO 30) were investigated. The hysteresis loops of the Zn_0.5_Cu_0.5_Fe_2_O_4_@rGO and Zn_0.5_Cu_0.5_Fe_2_O_4_ composite materials, tested at 298 K, are displayed in Fig. 7. The produced samples under research have their VSM characteristics values summarised in Table 1. In this context, it is well known that all produced samples exhibit magnetic behaviour characteristic of soft magnetic materials because of their tight hysteresis loops [48]. With a value of 37.264 emu/g, Zn_0.5_Cu_0.5_Fe_2_O_4_ exhibits the highest saturation magnetization (Ms). Through the diamagnetic rGO's increased loading to 30.163, 28.698, and 22.881 emu/g for MRGO10, MRGO20, and MRGO30, respectively, the values of Ms dropped. The successful creation of Zn_0.5_Cu_0.5_Fe_2_O_4_@rGO composites is indicated by this behaviour [26]. Moreover, when the proportion of magnetite nanoparticles rises, the magnetic remanence (Mr) value falls. Zn_0.5_Cu_0.5_Fe_2_O_4_'s Mr value was 1.569 emu/g; for MRGO 10, MRGO 20, and MRGO 30, respectively, this value dropped to 0.899, 0.719, and 0.546 emu/g. Reversible behaviour in hysteresis loops is lessened by the reduced coercivity values (< 100) shown by the magnetite nanoparticles and Zn_0.5_Cu_0.5_Fe_2_O_4_@rGO composites. Consequently, it is possible to classify the produced Zn_0.5_Cu_0.5_Fe_2_O_4_ and Zn_0.5_Cu_0.5_Fe_2_O_4_@rGO composites as superparamagnetic materials [26] and to attribute their significance to the nanocatalyst separation process. The superparamagnetic can make the Zn_0.5_Cu_0.5_Fe_2_O_4_ and Zn_0.5_Cu_0.5_Fe_2_O_4_@rGO nanocomposites more easily dispersible in the solution with negligible magnetic interactions between each other and avoid magnetic clustering[49]. The feature of the superparamagnetic nano-carriers is very bright not only in photocatalysis but also in drug delivery systems as, in site-specific cancer cells under an external magnetic field and many other applications [50].

**Table 1:** An overview of the magnetic characteristics of the Zn_0.5_Cu_0.5_Fe_2_O_4_, MRGO 10, MRGO 20, and MRGO 30

Magnetic properties	Saturation magnetization (M_s_) emu/g	Magnetic remanence (M_r_) emu/g	Coerctivity (H_c_) Oe
Zn_0.5_Cu_0.5_Fe_2_O_4_	37.264	1.569	32.56
MRGO 10	30.163	0.899	48.64
MRGO 20	28.698	0.719	50.51
MRGO 30	22.881	0.546	65.78

#### UV–visible spectrophotometric analysis

**Fig. 8:**
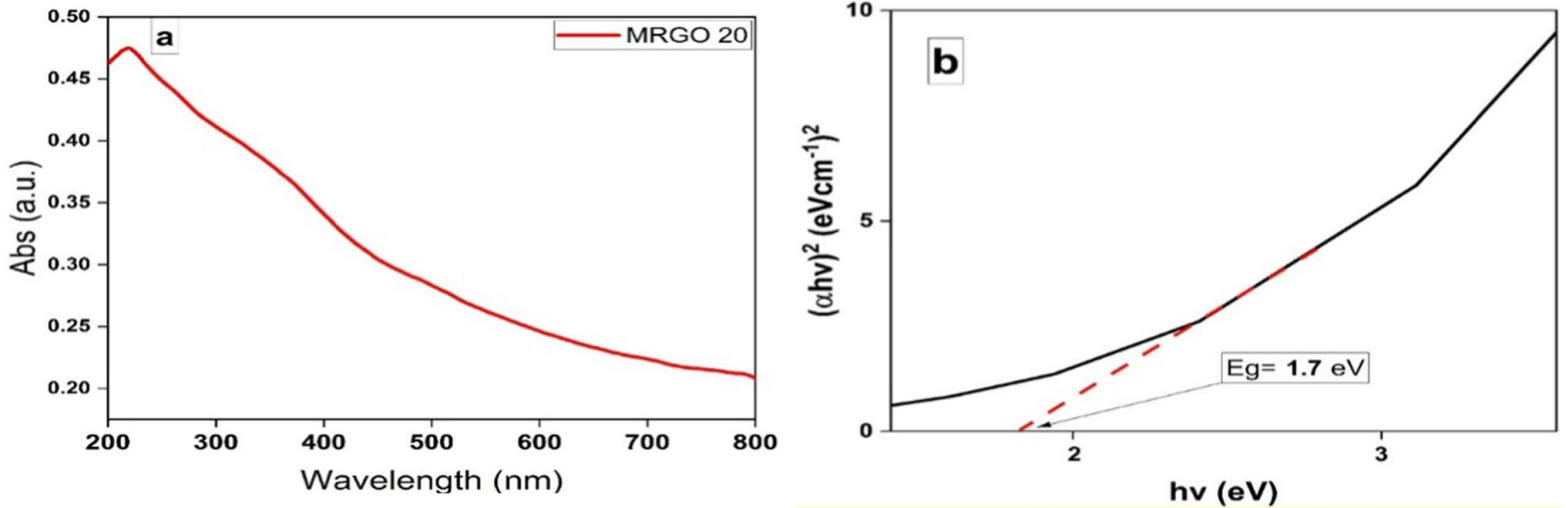
UV–visible absorption spectrum of the energy band gap of Zn_0.5_Cu_0.5_Fe_2_O_4_@rGO nanoparticles

Figure 8a and b displays the UV–visible absorption spectra of the of Zn_0.5_Cu_0.5_Fe_2_O_4_@rGO nanoparticles.

The UV–visible spectrum demonstrates that Zn_0.5_Cu_0.5_Fe_2_O_4_@rGO nanoparticles possesses a high absorptivity in the 250 nm wavelength region because of the part the hybridized Fe–d orbital plays, and has a band gab of 1.7 eV. The band gap of a photocatalyst plays a crucial role in its photocatalytic performance. The band gap energy determines the wavelength of light a photocatalyst can absorb. A lower band gap allows for absorption of longer wavelength light, including visible light. Wider band gap materials, like TiO2, primarily absorb UV light, which is a small portion of the solar spectrum. Narrower band gap materials can utilize a larger portion of the solar spectrum, including visible light. When a photocatalyst absorbs light with energy equal to or greater than its band gap, an electron is excited from the valence band to the conduction band, leaving a hole in the valence band. These photogenerated electron–hole pairs can participate in redox reactions, leading to the degradation of pollutants. A well-designed photocatalyst with lower band gap energy can promote charge separation, reducing recombination and increasing the lifetime of charge carriers.

### Photocatalytic potential of Zn_0.5_Cu_0.5_Fe_2_O_4_@rGO composites nanoparticles on MB

**Fig. 9:**
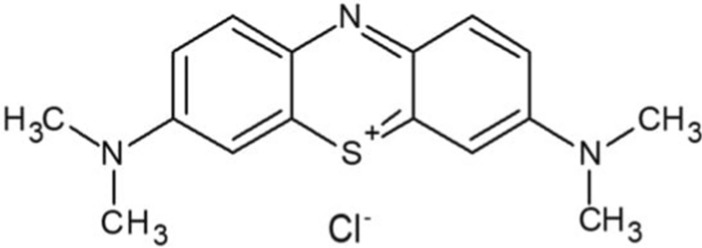
Methylene Blue Structural Formula

Under UV lamp light, the photocatalytic capability of the synthesized Zn_0.5_Cu_0.5_Fe_2_O_4_@rGO composite nanoparticles was investigated with regard to the removal of methylene blue (MB) as a contaminant. Often employed in a variety of sectors for a wide range of applications, including coloring textiles paper, wool, cotton, and coloring hair, and also use as a pharmaceutical, MB is a thiazine dye having strong carcinogenic qualities [51]. Other names for it are 3,7-bis(dimethylamino)phenothiazin-5-ium or methylthioninium chloride [9]. The formula for the MB structure is shown in Fig. 9 [44].

**Fig. 10:**
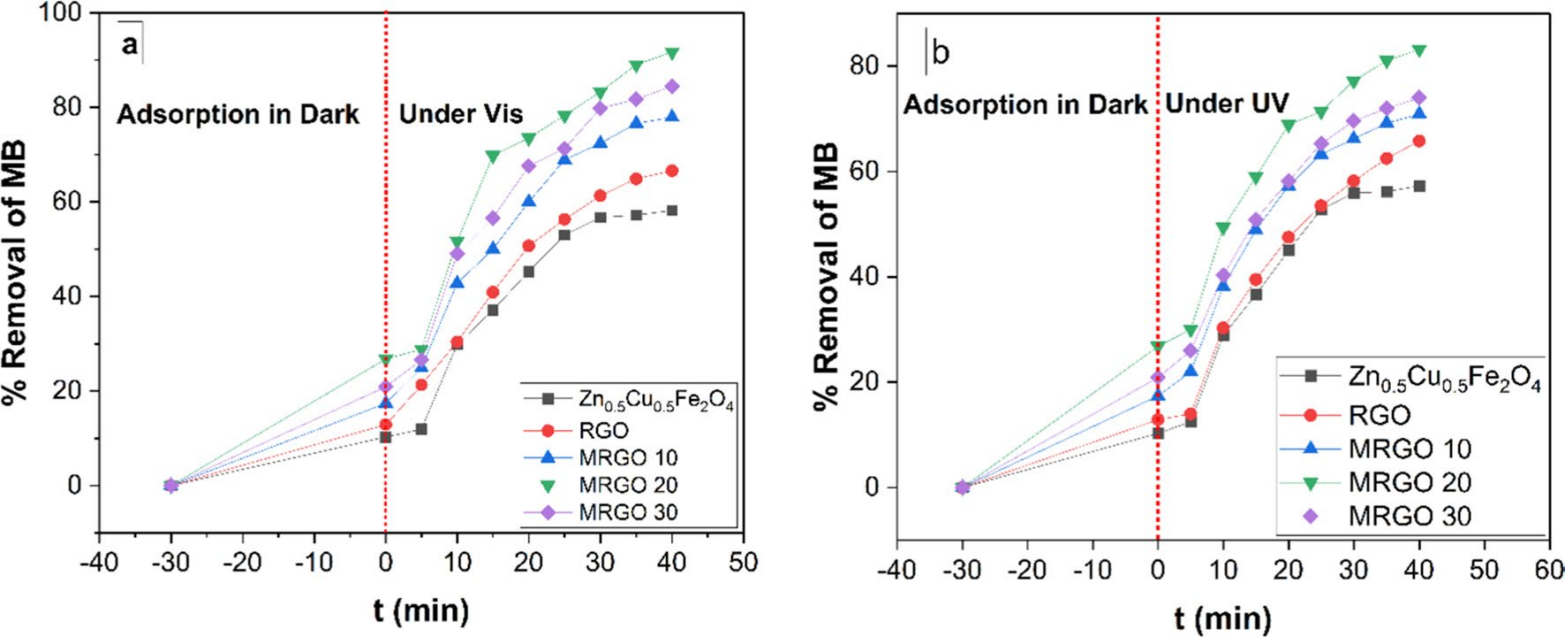
% Removal % of MB within 30 min adsorption activity in dark then 40 min by Zn_0.5_Cu_0.5_Fe_2_O_4_ NPs, RGO, MRGO 10, MRGO 20, and MRGO 30 **a** Photocatalysis under Visible light **b** Photocatalysis under UV

This section evaluated the Zn_0.5_Cu_0.5_Fe_2_O_4_NPs, RGO, MRGO 10, MRGO 20, and MRGO 30 the ability of photocatalysis to break down MB dye both when UV and visible light were present and when the light is absent. The concentration of MB dye was determined using at 664 nm wavelength with the UV–Vis DR5000 spectrophotometer [41, 52]. According to the results, the cationic MB dye for Zn_0.5_Cu_0.5_Fe_2_O_4_ NPs, RGO, MRGO 10, MRGO 20, and MRGO 30 samples all exhibit a slight degradation in the dark before being removed after 30 min. Additionally, Fig. 10a illustrates the time-dependent degradation of MB dye under visible light in the presence of Zn_0.5_Cu_0.5_Fe_2_O_4_ NPs, RGO, MRGO 10, MRGO 20, and MRGO 30. As shown in Fig. 10b, MB dye removal percentages were 58.2%, 66.6%, 77.9%, and 91.7%. The Zn_0.5_Cu_0.5_Fe_2_O_4_ NPs, RGO, MRGO 10, MRGO 20, and MRGO 30 on the other hand, showed relatively low photocatalytic degradation of MB dye under UV irradiation, of all the photocatalysts presented, the cocatalyst loaded MRGO has the highest photocatalytic activity for MB dye degradation under both UV and visible light, with respective values of about 57.3%, 65.8%, 70.9%, 83.2%, and 74%, respectively.

#### Effect of pH

**Fig. 11:**
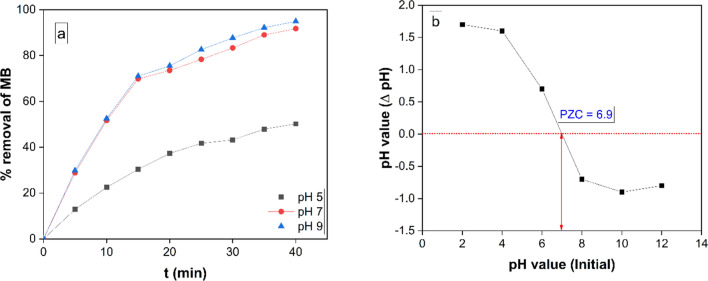
**a** 10 mL of MRGO 20 in 50 mL of 10 ppm of MB dye at 250C demonstrates how the percentage of MB removal increases over time at different solution pH levels (5.0, 7.0, and 9.0), and **b** putting the MRGO 20 PZC on display

One of the most important aspects of photocatalysis research is its sensitivity to solution pH [16, 53]. The effect of initial pH values of the MB solution was studied for 40 min under specified experimental conditions (10 mg of MRGO 20 nanocomposite, 50 ml of 10 ppm of MB dye solution, 25 °C). Figure 11a shows a graph showing the variation in MB removal (%) over time at three distinct solution pH levels (5.0, 7.0, and 9.0). At pH 9.0, the greatest MB Removal in equilibrium was noted, however pH 7.0 is also visible in the vicinity of pH 9.0. After 40 min, the percentage of MB dye removed with MRGO 20 with varying the pH values of 5.0, 7.0, and 9.0, were 52.4%, 91.7%, and 95.2% respectively.

To evaluate the point of zero charge (PZC) of the MRGO 20, 50 mL of 0.01 M NaCl solution was provided. Using HCl or NaOH, the pH of the solutions was adjusted to 2.0, 4.0, 6.0, 8.0, 10.0, and 12.0, then the samples were agitated for 48 h at 300 rpm. After MRGO 20 was magnetically separated, the pH values of the solutions were determined [54].

The pH at the zero-charge point was ascertained by plotting the beginning and ending pH values of MRGO 20, as seen in Fig. 11b. The pH of the PZC was determined equal to 6.90, as seen in Fig. 10b, where there is an insignificant difference between the pH readings at the start and finish. It demonstrates that the MRGO 20 photocatalyst has a photocatalyst's surface charge turns neutral at the pH of the PZC, and there is little to no electrostatic contact between the surface and the MB ions. The highest photocatalytic degradation of MB at pH 9.0, as shown in Fig. 10a, was clarified by the pH of the PZC value, which revealed that the pH of the PZC related MRGO 20 was 6.90. As a result, the positive charge of MB is attracted to the negative net surface charge of MRGO 20, boosting the photocatalytic degradation of MB. At pH = 7.0, the photocatalytic degradation of MB began to decrease. This is a result of the MB and MRGO 20 nanocomposite's opposing surface charges.

#### Effect of initial concentration

**Fig. 12:**
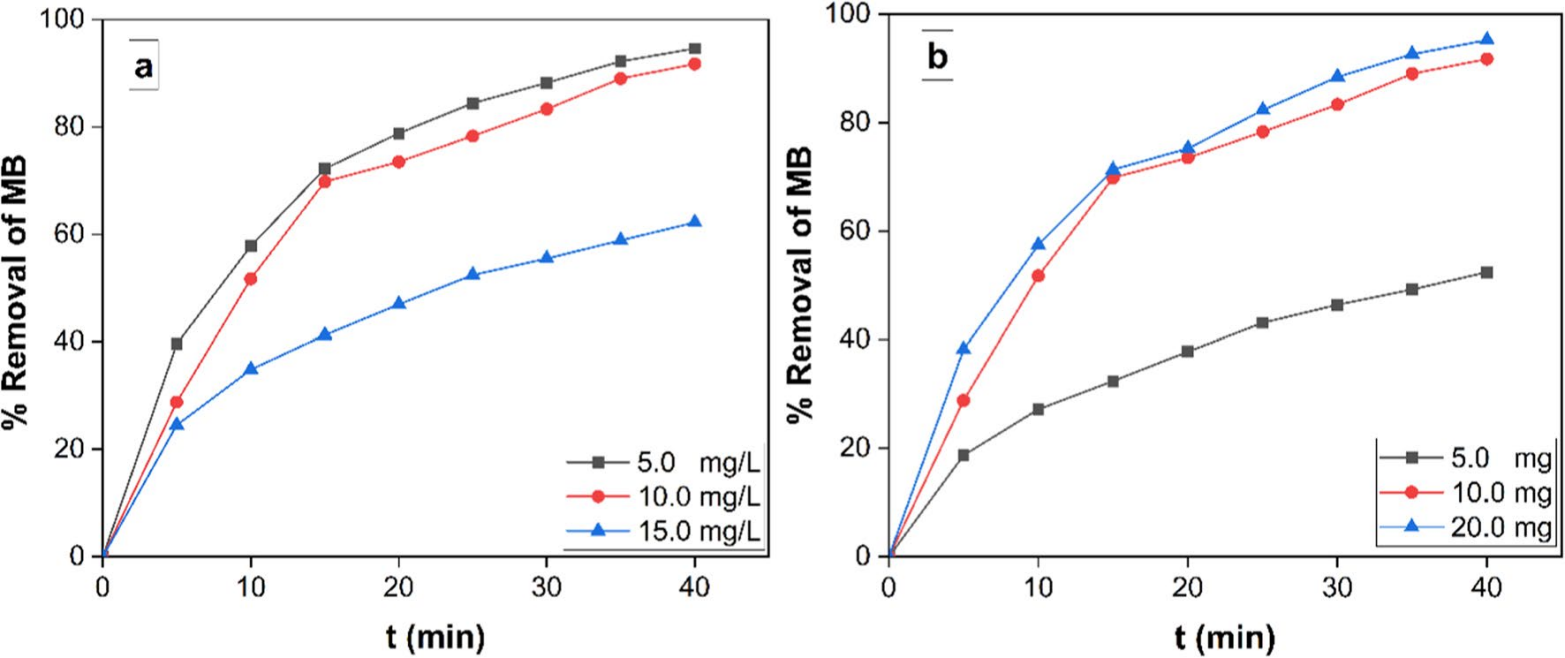
**a** 10.0 mg MRGO 20, changes in the percentage elimination with contact time at different initial MB concentrations (5, 10, and 15 ppm) at pH 7.0 and **b** the influence of 5, 10, and 20 mg photocatalyst dosages on the efficiency of MB removal (50 mL MB solution, 10 ppm, pH 9.0, 25 °C)

A change in MB ionic strength was made in order to evaluate its impact while keeping control over the other response parameters since the initial MB concentration was crucial to the elimination process [55]. Figure 12a depicts the differences in removal percentage as a function of contact time for the three different MB starting concentrations (5.0, 10.0, and 15.0 ppm). These findings show that when a synthesized MRGO 20 nanocatalyst is also present and exposed to visible light, MB can be efficiently eliminated, even at high initial concentrations. There is a negative relationship between the concentration of MB and the rate of degradation. After 40 min, the elimination percentage of MB dye was 94.6%, 91.7%, and 62.2% when MB dye was used at varying concentrations of 5.0 ppm, 10.0 ppm, and 20.0 ppm. Rostami et al. [56] confirmed that, ZnFe_2_O_4_–CuO–C_3_N_4_ ternary heterojunction nanophotocatalysts showed the highest photocatalytic activity of 95.84% for degradation of MO (5.0 ppm) in 80 min under UV light irradiation.

#### The impact of nanocomposite dose on degrading efficiency

Figure 12b illustrates that when changing the photocatalyst dosage between 5 and 20 mg in relation to a specific MB concentration (10 mg/L), it is feasible to examine the potential impact of an MRGO 20 dose on the efficiency of removing the MB under visible light. The results showed that increasing the amount of photocatalyst from 5 to 20 mg resulted in higher removal efficiency. After 40 min, the greatest photodegradation of MB using 20 mg/L of photocatalyst was 95.2% at an ambient temperature of 25 °C and a pH of 9.0. The improvement in removal efficiency reported with increased photocatalyst amount in the reaction may be explained by an increase in the photocatalyst's accessible active area or active sites in respect to the volume of the MB solution [57, 58]. Table 2 listed a different graphene-based photocatalysts for the photodegradation of different anionic and cationic dyes under UV and visible irradiation.

**Table 2:** Different graphene-based photocatalysts for the degradation of different anionic and cationic dyes

Photocatalyst	Targeted pollutant	Radiation/Light source	Degradation activity	Time (min)	Refs.
RGO-Fe_3_O_4_-TiO_2_	MB	Visible	94%	15	[59]
rGO/TiO2	MB	Visible	91.3%	30	[60]
AgNPs@GO	MB	Visible	92%	40	[61]
RGO/Fe_3_O_4_	MB	UV	95%	50	[62]
RGO-TiO_2_-CdO-ZnO-Ag	MB	UV	91%	15	[63]
γ –Fe_2_O_3_ @GO	MB	UV	90.06%	60	[64]
CuONPs@GO	MB	Visible	93.11	40	[61]
RGO-Ag_2_O/TiO_2_	Rhodamine B	UV	92.3%	70	[65]
TiO_2_/Ag/RGO	Rhodamine B	UV	92.9%	80	[66]
Ce_2_O_3_/BiVO_4_ @ RGO	MO	Visible	90%	120	[67]
CeVO_4_/BiVO_4_/RGO	MO	Visible	90%	120	[68]
Cu/Cu_2_O/RGO	MO	Visible	92%	30	[69]
RGO/g-C_3_N_4_	MO	Visible	92.3%	120	[70]
Ag_0.04_ZrO_2_/RGO	MO	Visible	87%	100	[71]
ZnS/RGO	MO	UV	70%	60	[72]
SnO_2_/RGO	MO	UV	84%	60	[73]
Ag/AgBr/RGO	MO	UV	90%	15	[74]
MRGO20	MB	Visible	95.2%	40	This Study

### Reuse and recycling

**Fig. 13:**
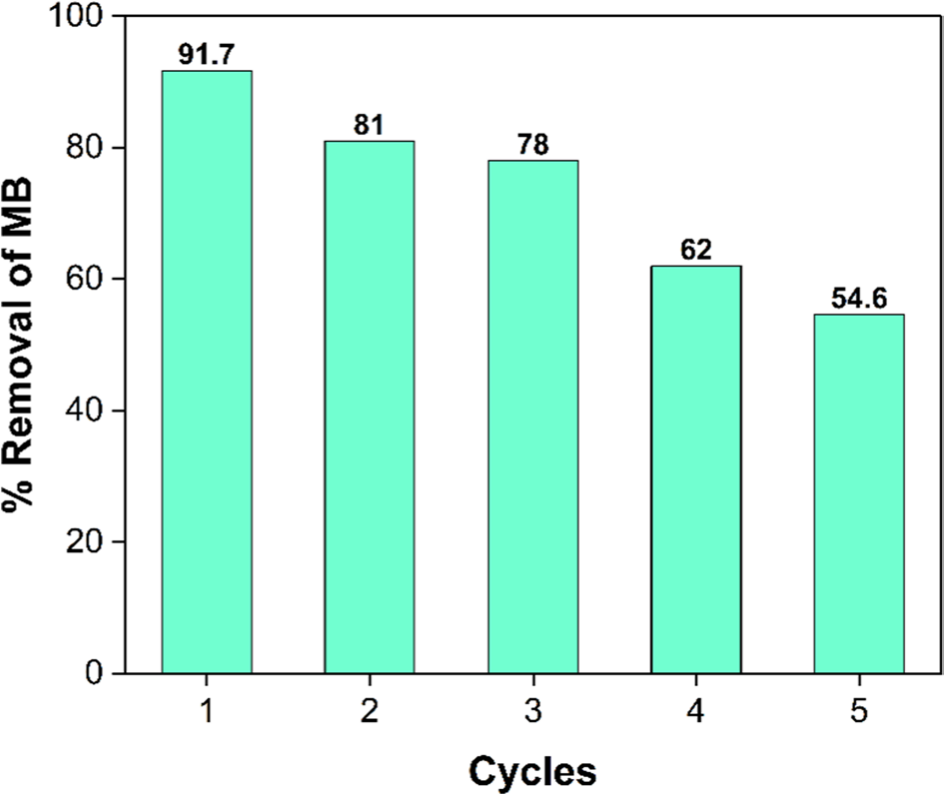
Recyclability of MRGO 20 for MB degradation by Visible light irradiation

Over time, waste management requires the extraction and reuse of photocatalysts employed in environmental remediation [75]. Consequently, the MRGO 20 sample demonstrated enhanced photocatalytic degradation efficiency and cost-effectiveness. Photocatalyst stability and long-term photocatalytic activity are both critical features. Reuse stability of a photocatalyst is essential to its industrial applications. MRGO 20 was collected by magnet, cleaned thrice with deionized water then let too dry in an oven for eight hours at 100 °C before being used in the next cycle. Further research was carried out, as indicated in Fig. 13, to examine the reuse stability of MRGO 20 in the photocatalytic reduction of MB dye under visible light irradiation. Tests for photocatalytic reuse were conducted in the same manner as for the assessment of photocatalytic activity previously described. The photocatalytic activity of MRGO 20 reduced to 54.6% after five cycles.

### Kinetic studies

**Fig. 14:**
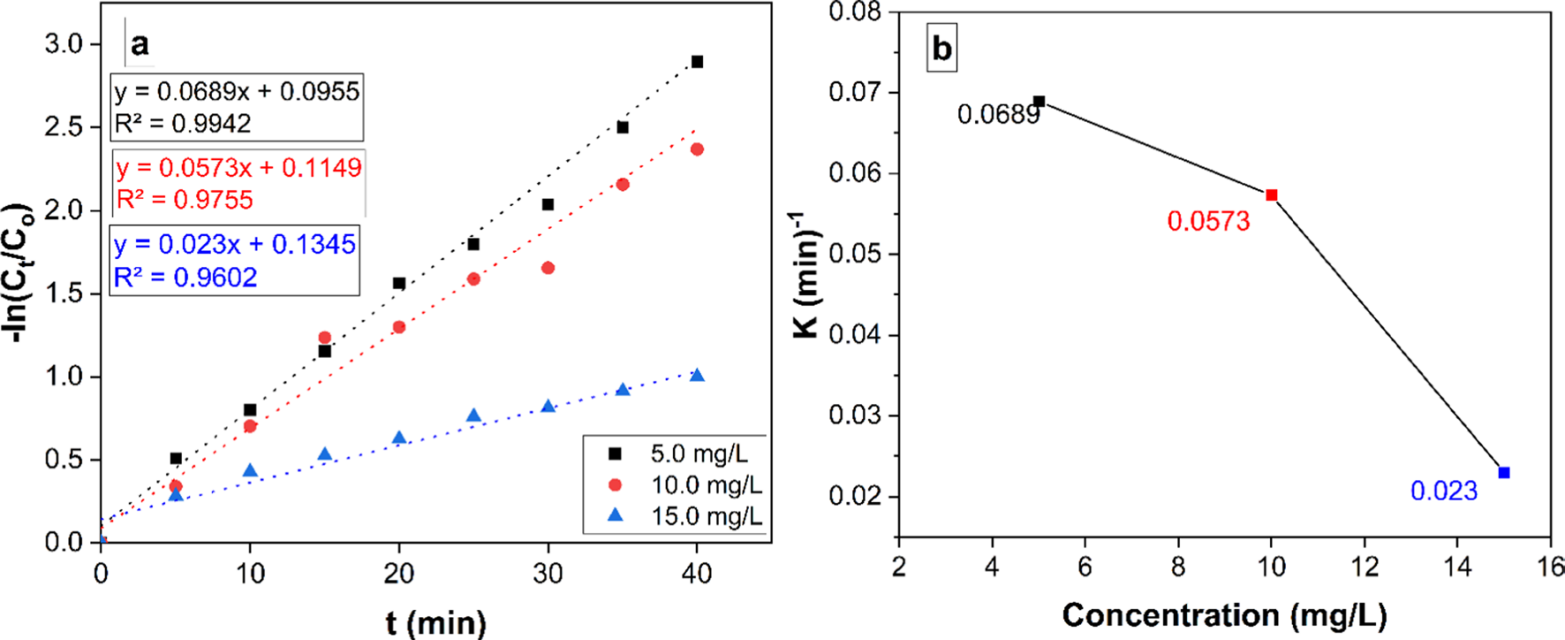
**a** With beginning concentrations of 10 ppm MB and 50 mL of 5, 10, and 15 mg catalyst doses, pseudo-first-order model data for MB degradation under visible light irradiation are supplied in kinetic form for a linear fit. **b** Explains how the initial MB concentration and the apparent pseudo-first order rate constants relate to each other

The rate of MB dye decay can be determined using the formula below:

2 $$-In\frac{Ct}{CO}=-Kt$$

where (t) is the removal time, (k) is the constant elimination rate, and (Ct and Co) are the appropriate MB dye starting and finishing concentrations. The relationship between (-ln Ct/Co) and time is depicted in Fig. 14a.

The findings demonstrate that pseudo-first-order rate rules control the reduction process's kinetics [76]. The pseudo-first-order rate constant decreases as the initial MB concentration rises, as seen in Fig. 14b. This emphasis on rate constants is consistent with the literature that was previously discussed [77].

#### Mechanism of photocatalysis of MB dye

Numerous studies [78] have validated the most likely approach, which is as follows: Changes in pH have an impact on three photodegradation mechanisms: Conduction band electrons explicitly reduce, while positive valence band holes explicitly oxidize, and assault by hydroxyl radicals. The presence of visible light will cause photocatalytic degradation because it produces electron–hole pairs on the surface of the employed MRGO 20 photocatalyst. The incorporation of rGO into Zn_0.5_Cu_0.5_Fe₂O₄ significantly enhances its photocatalytic activity through improved charge carrier dynamics [79]. rGO promotes effective electron–hole separation and mitigates electron recombination by providing a conductive pathway for electron transport, facilitating energy transfer, and increasing the overall reactivity of the photocatalytic system. This synergistic effect leads to a more efficient photocatalytic process, making the Zn_0.5_Cu_0.5_Fe₂O₄@rGO composite a promising candidate for applications such as pollutant degradation and solar energy conversion [80]. The holes' oxidative potential oxidises the reactive MB, producing breakdown products, or it forms hydroxyl radicals when it interacts with the –OH groups [78]. The following equations list the reactions that occur between MB and the used photocatalyst [81] (Eqs. 3–6).

3 $${\text{MRGO}}\;20 + {\text{h}}\upnu \to {\text{MRGO}}\;{20 }\left( {{\text{e}}^{-}_{{\text{(CB)}}} + {\text{h}}^{ + }_{{\text{(VB)}}} } \right)$$

4 $${\text{h}}^{ + }_{{({\text{VB}})}} + {\text{MRGO 2}}0 \to {\text{MRGO 2}}0^{ + } \left( {\text{Oxidation of the compound}} \right)$$

or

5 $${\text{h}}^{ + }_{{({\text{VB}})}} + {\text{OH}}^{ - } \to {\text{OH}}$$

6 $${\text{OH}}^{ \cdot } + {\text{MB}} \to {\text{Degradation}}\;{\text{products }}\left( {{\text{H}}_{{2}} {\text{O}} + {\text{CO}}_{{2}} } \right)$$

**Fig. 15:**
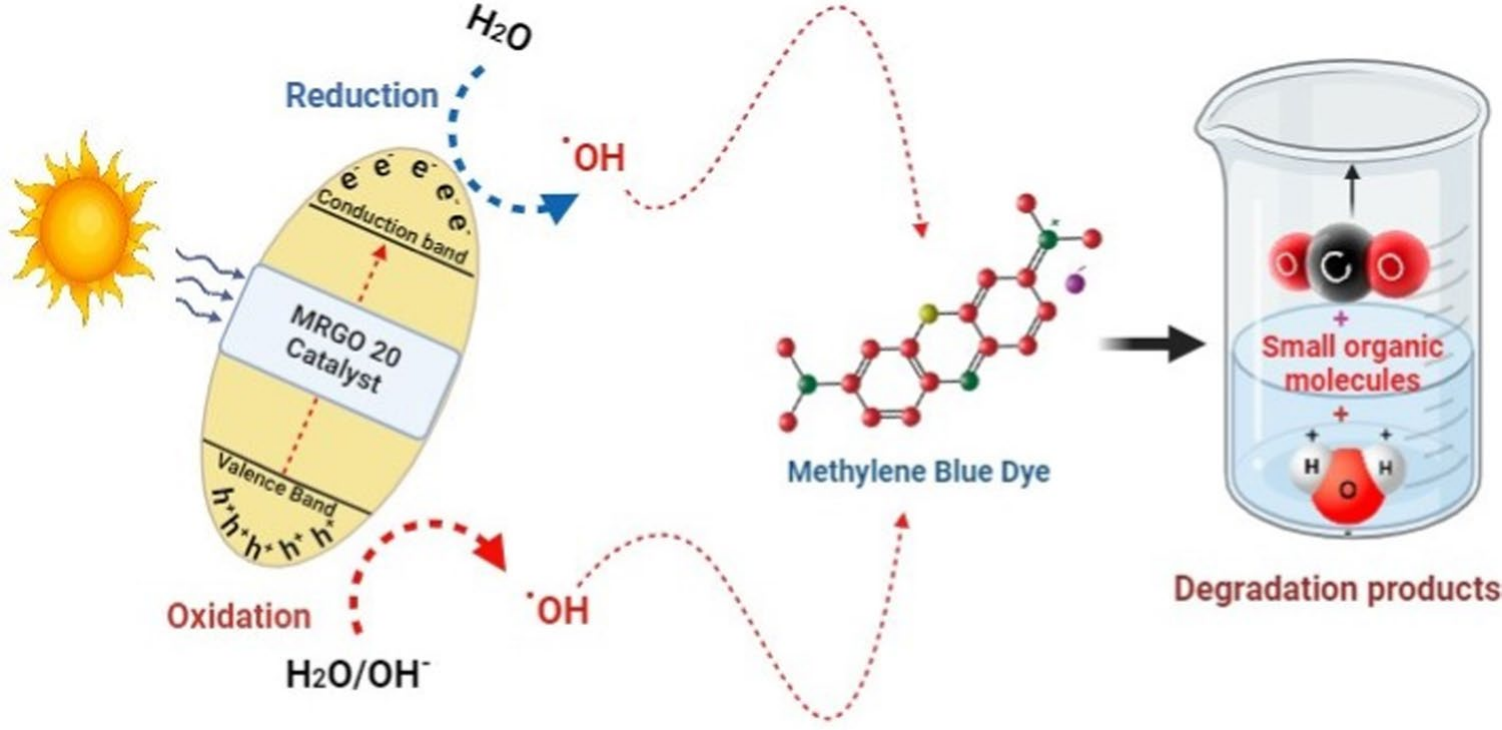
Potential photocatalytic reaction mechanism of MRGO 20 NPs nanocomposite-mediated photodegradation of Methylene Blue (MB)

Figure 15 represents the anticipated process of interaction between the produced nanocomposite and the MB. When the MRGO 20 photocatalyst is exposed to visible light, the semiconductor component (Zn_0.5_Cu_0.5_Fe₂O₄) absorbs photons, which excites electrons from the valence band to the conduction band, generating electron–hole pairs. Upon excitation, the generated electrons and holes can recombine if not efficiently separated. In the MRGO 20 photocatalyst, rGO acts as an electron sink, capturing the excited electrons and reducing the likelihood of recombination. The high electrical conductivity of rGO facilitates the movement of electrons away from Zn_0.5_Cu_0.5_Fe₂O₄, allowing them to participate in the subsequent reactions [82]. On the other hand, Methylene blue (MB) molecules are firstly adsorbed onto the surface of the composite catalyst. The large surface area of rGO enhances the adsorption capacity, allowing more MB molecules to meet the active sites on the catalyst. Once adsorbed, MB molecules can interact with the charge carriers. The holes generated in the valence band can oxidize MB, while the electrons can reduce oxygen molecules to form reactive oxygen species (ROS), such as hydroxyl radicals (•OH) and superoxide anions (•O_2_^−^). The generated hydroxyl radicals and other ROS can attack the MB molecules, breaking down the dye structure into smaller, less harmful compounds.

Because there are currently no published studies on the degradation of MB, additional research employing gas chromatography-mass spectrometry (GC–MS) and high-performance liquid chromatography (HPLC) is required to enhance the study of MB breakdown products.

## Conclusion

Co-precipitation was used to successfully create the nanocrystalline MRGO 20 nanocatalyst, which was then studied using optical and structural techniques. MB dye was used to assess the MRGO 20 nanocatalyst's photocatalytic efficiency. Furthermore, a number of factors, including the initial concentration of MB, the photocatalyst dosage, and the pH at which MB degrades, have been studied in relation to the removal potential's efficiency. In aqueous solutions, MRGO 20 NPs demonstrated a notable capacity for Methylene Blue photodegradation. After 40 min, the high photocatalytic efficacy effectively eliminated about 95.2% of the 10 ppm MB using 20 mg of MRGO 20 NPs at pH9 Visible light. The MRGO 20 nanocatalyst that was created shows promise for use in wastewater treatment. In the future work we will determine the photocatalytic activity of the prepared composites in degradation of real wastewater samples containing mixed dyes. Also, will determine the photocatalytic activity of the prepared composites in degradation of the wastes which exit from petroleum refining like (polyaromatic hydrocarbon, phenols and sulfur compounds).

## Data Availability

The data used to support the study's findings are accessible from the corresponding author upon request.
